# Cortisol concentration, pain and sedation scale in free roaming dogs treated with carprofen after ovariohysterectomy

**DOI:** 10.14202/vetworld.2017.888-894

**Published:** 2017-08-09

**Authors:** Katarina Nenadović, Marijana Vučinić, Brana Radenković-Damnjanović, Ljiljana Janković, Radislava Teodorović, Eva Voslarova, Zsolt Becskei

**Affiliations:** 1 Department of Animal Hygiene, Faculty of Veterinary Medicine, University of Belgrade, Serbia; 2 Department of Animal Protection, Welfare and Behaviour, University of Veterinary and Pharmaceutical Sciences Brno, Czech Republic; 3 Department of Animal Husbandry, Faculty of Veterinary Medicine, University of Belgrade, Serbia

**Keywords:** carprofen, cortisol, ovariohysterectomy, pain, sedation scale

## Abstract

**Background and Aim:**

One of the topic issues in animal welfare activities is the free roaming dog welfare especially in developing countries such as Serbia. The way of controlling population of free roaming dogs is their reproduction with the method of “Catch-Neuter-Release.” This complex process consists of capturing free roaming dogs in public areas, sterilizing, and returning them to the public area from which they were temporarily removed. Ovariohysterectomy present the period with a high intensity of stress reaction since many veterinarians in Serbia do not use analgesia for this group of dogs. The aim of this study was to compare the serum cortisol concentration before and after ovariohysterectomy and the level of post-operative pain and sedation in a group of free roaming female dogs treated with carprofen after surgical intervention and in a group with no treatment.

**Materials and Methods:**

The study was performed on a total of 20 female dogs under the program for free roaming dog control. Free-roaming dogs were captured in public areas by the communal animal hygiene service and were transported between 30 and 45 min to the clinic of a veterinary practice. Treatment began at 10:00 h on the next day and the bitches were kept in cages until they were returned to public locations from which they were temporarily removed to be sterilized. The G2 group received before closing the incision line carprofen in one dosage of 4 mg/kg given by subcutaneous injection into the scruff. Rescue protocol with carprofen was provided for G1 after 24 h following ovariohysterectomy same dosage as G2. Blood (2 ml) was collected from the cephalic vein of each dog in disposable plastic syringes, containing heparin (1:1000) 4 times: Before ovariohysterectomy, 30, 120 min and 24 h following ovariohysterectomy. Cortisol concentration was determined by enzyme-linked immunosorbent assay. The multifactorial pain and sedation scale were used for the assessment of pain and sedation.

**Results:**

In both groups, the lowest values of serum cortisol concentration were obtained before ovariohysterectomy. Cortisol levels in both groups were significantly higher (p<0.01) 30 and 120 min after ovariohysterectomy and showed a decreasing trend toward the end of the observation period (24 h). The results obtained 15 and 30 min after the surgical intervention have revealed a statistically significant difference between the groups (p<0.05) showing that female dogs treated with carprofen had a lower value on the pain scale and a higher value on the sedation scale compared to the group with no treatment.

**Conclusion:**

Carprofen provides both a restful consequence of sedation and a rapid return to a more normal physiological and behavioral state in dogs after ovariohysterectomy.

## Introduction

Surgical procedures represent a major source of stress for the animal, due to the surgery itself and various associated elements, such as pain, analgesia, and anesthesia induced dysphoria, human handling, and confinement in a hospitalization unit [1-6]. Surgical stress and stress associated with related procedures have been evaluated in dogs using different markers. Cortisol and behavioral analysis have often been used [1,7-15]. Ovariohysterectomy is one of the most commonly practiced surgical interventions in veterinary medicine [16], including animal birth control programs thus securing management of free-roaming dog populations. It is considered to be an intervention of medium level pain intensity. Post-operative pain is inevitable after ovariohysterectomy. Therefore, a suitable selection of analgesic drugs for the post-operative period is highly important for animal welfare. However, many veterinarians in Serbia do not treat free-roaming bitches with analgesics in the perioperative period of the intervention since pain management protocol for this group of dogs does not exist.

Increased knowledge, changing attitudes, and greater sensibility for animal welfare have increased the willingness to treat pain in veterinary practice. Besides, an appropriate post-operative pain therapy with few or even no side effects has been proven to result in a better and more satisfactory recovery. Therefore, all animals undergoing surgical procedures require pain relief after surgery to overcome the deleterious physiological effects of post-operative pain and to address humane and ethical concerns [17].

Carprofen is an nonsteroidal anti-inflammatory drug of the carbazole group and is a propionic acid derivative. It is especially highly effective in controlling the pain caused by degenerative joint diseases and is also quite effective in eliminating post-operative soft tissue and orthopedic pain [18-24].

This study aimed to determine the effect of carprofen used in the surgical intervention (ovariohysterectomy) on serum cortisol levels, pain and sedation scale in free roaming dogs.

## Materials and Methods

### Ethical approval

Research protocols using animals followed guidelines of the Ethical Committee of the Faculty of Veterinary Medicine, University of Belgrade, Serbia, as well as EU Directive 2010/63/EU for animal experiments. The study was reviewed and approved by Animal Ethical Committee of the Faculty of Veterinary Medicine, University of Belgrade, Serbia (01 - 18/18).

### Animals and treatment

The study was performed on a total of 20 female dogs under the program for free roaming dog control, which underwent ovariohysterectomy at the Faculty of Veterinary Medicine, University of Belgrade. All bitches included in the experiment were approximately the same age (2-4 years old) and the same body weight (20±2 kg). Free-roaming dogs were captured in public areas by the communal animal hygiene service and were transported between 30 and 45 min to the clinic of a veterinary practice. The captures and transport of dogs were performed according to current Serbian Animal Welfare Law 41/2009. The drivers and the travel circuit was the same for all days. Usually, a departure travel took place between 08:30 and 09:30 h. Dogs were individually housed in typical (1 m × 1 m × 1 m) cages with possibility of visual, olfactory and auditory contact between animals. In new environment, water was available *ad libitum*. Food was withheld for 8-12 h before anesthesia. Treatment began at 10:00 h on the next day and the bitches were kept into cages until they were returned to public locations from which they were temporarily removed to be sterilized.

The health status of dogs was assessed before the operation procedure. To be included in the study, the dogs were required to have a physical examination and pre-operative blood analysis, i.e., complete blood count and serum biochemistry profile. Only dogs with no abnormal signs of fever or visible acute and chronic disorders were operated. The dogs were randomly allocated to one of two groups of 10 each. Group 1 dogs were not treated with nonsteroid analgesic drugs (G1). In Group 2, dogs were treated with nonsteroid anti-inflammatory analgesic on the completion of the surgical intervention (G2). All of the dogs were submitted to identical pre-operative and operative/anesthesia protocol applied at the Surgical Department of the Faculty of Veterinary Medicine in Belgrade. Premedication included atropine sulfate administered subcutaneously in dosage from 0.02 to 0.04 mg/kg and acepromazine administered intramuscularly in a dosage of 0.03 mg/kg. After 15 min, for injectable anesthesia diazepam in a dosage of 0.2-0.5 mg/kg and 0.5 mg/kg of 10% ketamine-hydrochloride were administered intravenously. 10% ketamine-hydrochloride (15 mg/kg) was administered intramuscularly for maintenance general anesthesia. During the surgical procedure until waking up from anesthesia, the dogs were infused with saline solution with 5% glucose. In addition, the G2 group received before closing the incision line carprofen (propionic acid derivate; 6-chloro-alpha-methyl-9H-carbazole-2-acetic acid; Rimadyl, Pfizer) in a dosage of 4 mg/kg given by subcutaneous injection into the scruff. The dosages used are based on commonly used clinical dosages and manufacturer’s recommendations. Ovariohysterectomy procedure performed with traditional open surgery (removal of uterus and ovaries). The skin is incised along the linea alba, that is, the sheath of the rectus abdominus, starting from the umbilicus and ending a few centimeters in front of the pubis. To exclude the possibility of influence operation technique on results, the all operation procedures were performed by the same veterinary surgeon. After surgery, all dogs were returned into the cages.

For ethical reasons, a rescue protocol with carprofen was provided for G1 after 24 h following ovariohysterectomy.

### Hematological analysis

Blood (2 ml) was collected from the cephalic vein of each dog in disposable plastic syringes, containing heparin (1:1000) 4 times: Before ovariohysterectomy, 30, 120 min and 24 h following ovariohysterectomy. The blood was centrifuged at 3000 rpm for 5 min and serum was separated and stored at −20°C until assayed. Serum cortisol concentration was determined by enzyme-linked immunosorbent assay described by Ginel *et al*. [25].

### Measures of pain and sedation level

The degree of post-operative pain/discomfort was assessed 15, 30, 60, 120 min and 24 h after ovariohysterectomy according to the multifactorial pain scale which has been successfully used in several clinical studies [26-34], consisting of a number of simple descriptive scale values relating to the particular aspects of behavior that may be associated with pain (Table 1) by a one of the trained authors. The sum of established scores for each dog were classified from 0 to 9 based on the state of animal as following: 0 - Complete analgesia, with no overt signs of discomfort and no reaction to firm pressure, 1-3: Good analgesia, with no overt signs of discomfort but reactive to firm pressure, 4-6: Moderate analgesia, with some overt signs of discomfort which were made worse by firm pressure, 7-9: Poor or no analgesia, with obvious signs of persistent discomfort worsening by firm pressure [30].

**Table-1:** Multifactorial pain scale [26].

Behavior and description of experience	Pain score
Sings of crying and whimpering	
Quiet, peaceful	0
Occasional vocalization	1
Vocalizing most of the time, animal cannot be calmed down patting or gentle talking	2
Movements	
Comfortable at moving, otherwise quiet	0
Occasionally changing the position looking for more comfortable one, but not interfering with the wound	1
Changing the position most of the time, looking to the surgical wound, trying to lick it	2
Restlessness and discomfort	
Peaceful, but interested in surroundings	0
Moderately restless	1
Anxious, stressed	2
Response to the firm pressure applied adjacent to the surgical wound	
No reaction to firm pressure in terms of vocalization turning the head toward the wound and trying to bite the assessor, moving away of the assessor, anxiety	0
Reacts to firm pressure when repeated 4 times	1
Reacts to firm pressure when repeated 3 times	2
Reacts to firm pressure immediately or when the pressure to the wound is repeated twice	3

Post-operative sedation was assessed by observing the dog’s posture, the degree of mental alertness and the ability to stand and walk at 15, 30, 60, 120 min and 24 h after ovariohysterectomy. Sedation was scored according to the discontinuous scoring system using a simple numerical rating scale as follows: 0 - Fully alert and able to stand and walk, 1 - Alert and able to maintain sternal recumbency, not stable when trying to walk, 2 - Drowsy and able to maintain sternal recumbency but unable to stand, and 3 - Fast asleep [30].

### Statistical analysis

Data were statistically processed and analyzed by the GraphPad Prism 5.0 software. Distribution of the cortisol concentration indicator, pain and sedation scores were tested by the Kolmogorov–Smirnov distribution fitting test, which showed that distribution was not normally distributed. Median pain and sedation scores and cortisol concentration were compared between and within groups at each of the assessment points. These values were compared using the Kruskal–Wallis test. Where statistical differences were noted, pair-wise comparisons were performed using the Mann–Whitney U-test.

## Results

**Figure-1:**
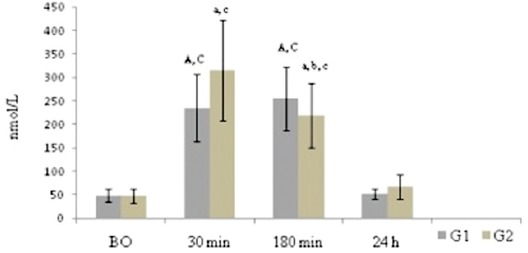
Serum cortisol concentration (nmol/L) in G1 and G2 groups (n=10 per group). ^A,a^Differences compared to BO; ^B,b^Differences compared to 30 min; ^C,c^Differences compared to 24 h; BO - Before the ovariohysterectomy. Capital letters indicate significant difference (p<0.01) between time points in G1. Small letters indicate significant difference (p<0.01) between time points in G2.

The obtained study results indicated that in both groups, serum cortisol concentration after ovariohysterectomy was significantly higher (p<0.01) in comparison with baseline concentration. Furthermore, 24 h after ovariohysterectomy significantly a lower (p<0.01) cortisol concentration was recorded in both groups compared to concentrations 30 and 120 min after ovariohysterectomy. Cortisol concentration in G2 group was significantly lower (p<0.01) and in G1 group was higher 120 min after ovariohysterectomy in relation to 30 min, which is shown in Figure-1. There were no differences between groups for cortisol concentration.

The median (range) values of individual behaviors (included in multifactorial pain scale and sedation scale) of each group are shown in Table 2.

**Table-2:** Pain and sedation score presented as median (range) at each of the assessment points in G1 and G2.

Parameter	Group	Pain scores median (range)				

		15 min	30 min	60 min	120 min	24 h
Sings of crying and whimpering	G1	0 (0-0)	0 (0-0)	0 (0-0)	0 (0-0)	0 (0-0)
	G2	0 (0-0)	0 (0-0)	0 (0-0)	0 (0-0)	0 (0-0)
Movements	G1	0 (0-2)	0 (0-1)	0 (0-2)	0 (0-1)	0 (0-0)
	G2	0 (0-0)	0 (0-0)	0 (0-1)	0 (0-1)	0 (0-0)
Restlessness and discomfort	G1	0.5 (0-1)	0.5 (0-1)	0 (0-2)	0.5 (0-1)	0.5 (0-1)
	G2	0 (0-1)	0 (0-1)	0 (0-1)	0 (0-0)	0 (0-0)
Response to the firm pressure applied adjacent to the surgical wound	G1	0 (0-3)	0 (0-1)	0 (0-1)	0 (0-1)	0 (0-3)
	G2	0 (0-0)	0 (0-0)	0 (0-0)	0 (0-0)	0 (0-0)
Total	G1	1.5 (0-4)*	1.5 (0-3)*	1.5 (0-4)	1 (0-2)	0 (0-3)
	G2	0 (0-1)	0 (0-1)	0 (0-1)	0 (0-1)	0 (0-0)

**Parameter**	**Group**	**Sedation score median (range)**				

		**15 min**	**30 min**	**60 min**	**120 min**	**24 h**

Total	G1	2 (1-3)^a^	1.5 (1-3)^a^	1 (0-2)	0.5 (0-2)	0 (0-0)
	G2	3 (2-3)^ab^*	3 (1-3)^ab^*	1 (0-3)	1 (0-1)	0 (0-0)

a Differences compared to 120 min.

b Differences compared to 24 h within the group (p<0.05).

* Significant differences between groups (p<0.05)

### Sings of crying and whimpering

In both groups (G1 and G2) at each of the assessment points, dogs were not vocalized.

### Movements

In G1, Score 1 was obtained in 2 dogs at 15, 60 and 120 min and 4 dogs at 30 min after surgery. Score 2 was obtained in 1 dog at 15 and 60 min after surgery. In G2, Score 1 was noticed in 3 dogs at 60 and 120 min following ovariohysterectomy. In both groups, at 24 h all dogs were comfortable at moving, otherwise quiet.

### Restlessness and discomfort

In G1, Score 1 was evident in 5 dogs at 15, 30, 120 min and 24 h and 3 dogs at 30 min after surgery. One dog at 60 min had Score 2. In G2, 2 dogs at 15 min and 1 dog at 30 and 60 min following ovariohysterectomy had Score 1.

### Response to the firm pressure applied adjacent to the surgical wound

In G1, Score 1 was detected in 2 dogs at 30 and 120 min and 3 dogs at 60 min. Score 3 was detected in 1 dog at 15 min and 2 dogs at 120 min after surgery. In G2, all dogs at each of the assessment point had no reaction to firm pressure in terms of vocalization, turning the head toward the wound and trying to bite the assessor, moving away of the assessor, anxiety.

There were no differences between and within groups for individual behaviors which is shown in Table 2.

As presented in Table 2, the highest value of pain intensity in G1 group was determined 15 and 60 min after the surgery and the lowest 24 h after the surgery. In the G2 group, the highest pain was noticed 60 min after the surgery, while total analgesia occurred 24 h following the surgery. Comparing the pain intensity between the groups, a significantly greater value (p<0.05) was found in G1 group at 15 and 30 min after the surgery. Evaluation of pain intensity in other observation periods revealed nonsignificant differences between the groups.

### Sedation score

In G1, Score 1 was detected in 4 dogs at 15 min, 5 dogs at 30 min, 6 dogs at 60 min and 3 dogs at 120 min. Three dogs had Score 2 at 15 and 30 min after surgery and 2 dogs at 60 and 120 min. Score 3 was obtained in 3 dogs at 15 min and 2 dogs at 30 min. In G2, 7 dogs had Score 1 at 60 and 120 min and 1 dog at 30 min. Score 2 was noticed in 3 dogs at 15 and 30 min and 1 dog at 60 min while Score 3 was obtained in 7 dogs at 15 min, 6 dogs at 30 min and 1 dogs at 60 min following ovariohysterectomy. After 24 h, all dogs were fully alert and able to stand and walk.

As presented in Table 2, sedation scores obtained at 15 and 30 min in both groups were significantly higher (p<0.05) compared with other assessment points. The G2 was significantly more (p<0.05) sedated compared to G1 at 15 and 30 min after the surgery.

## Discussion

In this study, significant rise of the serum cortisol level was obtained in the post-operative period compared to the pre-operative period in both groups of observed dogs. Similar results have been documented in the previous studies [1,10,11,34-37]. Significantly higher cortisol concentrations were noticed 30 and 120 min after ovariohysterectomy in both groups compared to the pre-operative period indicated the activity of the hypothalamic-pituitary-adrenal gland. In the G2, the level of cortisol was lower 120 min after ovariohysterectomy compared with cortisol concentration 30 min after ovariohysterectomy. This result might be due to efficiency of carprofen in controlling post-operative pain as confirmed in previous studies [19,20,30,38,39]. On the other hand, the increased average level of cortisol level obtained 120 min after ovariohysterectomy indicating the presence of pain and acute stress in G1 group. These findings suggest that we can only assume that stress exists since cortisol is a very unspecific hormone and could be altered due to stress related to pain, but also fear, cold or even the anesthesia itself. The cortisol concentration did not show the clear differences between groups that might have been expected by the results for the pain and sedation score. Individual differences like the effects of an unknown breed or earlier life experiences may further attribute to variability in cortisol concentration and stress response.

Tissue injury leads to the activation of nociceptive and inflammatory responses that are often associated with pain and hyperalgesia and behavioral changes [1,8,10,11,40]. Changing of behavior in the post-operative period is a consequence of unpleasant physical and emotional experiences such as discomfort, unpleasant, insecurity and unhappiness, hunger, thirst, space and partial social isolation, fear, anxiety, stress, pain, inability to control the living conditions, the inability to predict the course of events, and the manifestation of natural forms of behavior. Observation of animal behavior is a noninvasive method that allows fast control of stress [41-43].

According to the study results, significantly lower pain scores were found at 15 and 30 min after ovariohysterectomy in G2 group in comparison with G1 group. According to Seliškar *et al*. [30], pain score was lower in the group treated with carprofen, but the difference was nonsignificant. The different level of pain recorded between the G1 and G2 group in this study could be ascribed to the analgesic effect of carprofen in treated animals. Low values of pain obtained in our study could be also related to the fact that ketamine, which was used for anesthesia, has hyperalgesia preventive effects lasting 10-12 h post administration [39]. Pain assessment performed immediately after surgery may be difficult to interpret because of the changes associated with recovery from anesthesia, such as residual sedation or shivering [44]. The intensity of pain may be higher than behavioral changes might suggest. Response to the firm pressure applied adjacent to the surgical wound was expressed only in G1 at each of the assessment points while G2 was without reaction. Drug-induced sedation can mask the presence of pain by dampening the overt signs even though the pain may not be attenuated, and is therefore important to distinguish sedation and analgesia [30]. However, the same group (G2) had a significantly higher sedation score compared with G1 at 15 and 30 min following ovariohysterectomy. Dogs from G1 were occasionally changed body position at 15 min while dogs from G2 performed this action at 60 min following ovariohysterectomy which may be related to the fact that dogs from G1 had lower sedation score. This effect might be due to the mild sedation caused by the carprofen, with the dogs tending to change position less frequently. On other hand, the possibility exists that increased changing body position in G1 dogs was due to restlessness to pain or increased noise sensitivity. These results are in accordance with the previous results [16,45]. Therefore, the animals were not able to express the behavioral changes that occur during painful stimuli such as vocalization, licking and chewing the painful place. It suggests that changes in the behavior itself are not being enough for the evaluation of post-operative pain and points to the importance of combining biochemical and behavioral parameters.

The pain intensity increased slightly after 60 min of observation in both groups of dogs. This could be attributed to an abrupt decrease of sedation score value observed 60 min after surgery in both groups which relate to the termination of sedation and return of consciousness when animals probably face the greatest challenge due to post-operative stress and inflammation.

## Conclusion

Carprofen provides both a restful consequence of sedation and a rapid return to a more normal physiological and behavioral state in dogs after ovariohysterectomy. Improvement and implementation of the usage of pain and sedation scale is an important step toward establishment animal welfare in perioperative period of ovariohysterectomy for free roaming dogs. Appropriate individual cages for housing in clinic of a veterinary practice, handling with animals, using appropriate anesthesia and analgesia to mitigate pain and faster recovery animals and reducing disphorie are just some of the critical control points that can improve the welfare of free roaming dogs undergoing surgical procedures.

## Authors’ Contributions

KN research creation and design; MV critical revision of the manuscript as for important intellectual content; BRD, LJ, RT analysis and interpretation of data; EV statistical analysis and manuscript drafting; ZB data acquisition. All authors read and approved the final manuscript.

## References

[1] Hansen B.D, Hardie E.M, Caroll G.S (1997). Physiological measurements after ovariohysterectomy in dogs: What’s normal?. Appl. Anim. Behav. Sci.

[2] Jirkof P, Cesarovic N, Rettich A, Arras M (2013a). Housing offemale mice in a new environment and its influence on post-surgical behaviour and recovery. Appl. Anim. Behav. Sci.

[3] Mellor D.J, Cook C.J, Stafford K.J, Moberg G.P, Mench J.A (2000). Quantifying some responses to pain as a stressor. The Biology of Animal Stress.

[4] Hekman J.P, Karas A.Z, Sharp C.R (2014). Psychogenic stress in hospitalized dogs: Cross species comparisons, implications for health care, and the challenges of evaluation. Animals.

[5] Sarah A, Robinsona S.J (2015). Reducing the stress of drug administration: Implications for the 3Rs. Sci. Rep.

[6] Horta R.S, Figueiredo M.S, Lavalle G.E, Costa M.P, Cunha R.M, Araujo R.B (2015). Surgical stress and postoperative complications related to regional and radical mastectomy in dogs. Acta Vet. Scand.

[7] Beerda B, Schilder M.B, van Hooff J.A.R (1997). Manifestation of chronic and acute stress in dogs. Appl. Anim. Behav. Sci.

[8] Beerda B, Schilder M.B, van Hooff J.A.R (1998). Behavioural, saliva cortisol and heart rate responses to different types of stimuli in dogs. Appl. Anim. Behav. Sci.

[9] Feldman E.C, Nelson R.W, Canine hyperadrenocorticism (2004). Canine and Feline Endocrinology and Reproduction.

[10] Siracusa C, Manteca X, Cerón J (2008). Perioperative stress response in dogs undergoing elective surgery: Variations in behavioural, neuroendocrine, immune and acute phase responses. Anim. Welfare.

[11] Väisänen M.N, Raekallio M, Kuusela E (2002). Evaluation of the perioperative stress response in dogs administered medetomidine or acepromazine as part of the preanesthetic medication. Am. J. Vet. Res.

[12] Hekman J.P, Karas A.Z, Dreschelb N.A (2012). Salivary cortisol concentrations and behavior in a population of healthy dogs hospitalized for elective procedures. Appl. Anim. Behav. Sci.

[13] Srithunyarat T, Höglund O.V, Hagman R, Olsson U, Stridsberg M, Lagerstedt A.S, Pettersson A (2016). Catestatin, vasostatin, cortisol, temperature, heart rate, respiratory rate, scores of the short form of the Glasgow composite measure pain scale and visual analog scale for stress and pain behavior in dogs before and after ovariohysterectomy. BMC Res. Notes.

[14] Michelsen J, Heller J, Wills F, Noble G.K (2012). Effect of surgeon experience on postoperative plasma cortisol and C-reactive protein concentrations after ovariohysterectomy in the dog: A randomised trial. Aust. Vet. J.

[15] Fazio E, Medica P, Cravana C, Pupillo A, Ferlazzo A (2015). Effects of ovariohysterectomy in dogs and cats on adrenocortical, haematological and behavioural parameters. Acta Sci. Vet.

[16] Fox S.M, Mellor D.J, Stafford K.J, Lowoko C.R, Hodge H (2000). The effects of ovariohysterectomy plus different combinations of halothane anaesthesia and butorphanol analgesia on behaviour in the bitch. Res. Vet. Sci.

[17] Dzikiti T.B, Joubert K.E, Venter L.J (2006). Comparison of morphine and carprofen administered alone or in combination for analgesia in dogs undergoing ovariohysterectomy. J. S. Afr. Vet. Assoc.

[18] Erol M, Izci C (2011). Postoperative analgesic effects of carprofen following osteotomy and laparotomy in dogs. J. Anim. Vet. Adv.

[19] Laredo F.G, Belda E, Murciano J, Escobar M, Navarro A, Robinson K.J (2004). Comparison of the analgesic effects of meloxicam and carprofen administered preoperatively to dogs undergoing orthopaedic surgery. Vet. Rec.

[20] Lascelles B.D.X, Cripps P.J, Jones A, Waterman-Pearson A.E (1998). Efficacy and kinetics of carprofen, administered preoperatively or postoperatively, for the prevention of pain in dogs undergoing ovariohysterectomy. Vet. Surg.

[21] Leece E.A, Brearley J.C, Harding E.F (2005). Comparison of carprofen and meloxicam for 72 hours following ovariohysterectomy in dogs. Vet. Anaesth. Analg.

[22] Slingsby L.S, Murison P.J, Goossens L, Engelen M, Waterman-Pearson A.E (2006). A comparison between pre-operative carprofen and a long-acting sufentanil formulation for analgesia after ovariohysterectomy in dogs. Vet. Anaesth. Analg.

[23] Delgado C, Bentley E, Hetzel S, Smith L.J (2014). Carprofen provides better post-operative analgesia than tramadol in dogs after enucleation: A randomized, masked clinical trial. J. Am. Vet. Med. Assoc.

[24] Kurum B, Pekcan Z, Kalender H, Kumandas A, Mutan O.C, Ertuğrul E (2013). Comparison of propofol-remifentanil and propofol-fentanyl anesthesia during ovariohysterectomy in dogs. Kafkas Univ. Vet. Fak.

[25] Ginel P.J, Pérez-Rico A, Moreno P, Lucena R (1998). Validation of a commercially available enzyme-linked immunosorbent assay (ELISA) for the determination of cortisol in canine plasma samples. Vet. Res. Commun.

[26] Morgaz J, Muñoz-Rascón P, Serrano-Rodríguez J.M, Navarrete R, Domínguez J.M (2014). Effectiveness of pre-peritoneal continuous wound infusion with lidocaine for pain control following ovariohysterectomy in dogs. Vet. J.

[27] Bosmans T, Gasthuys F, Duchateau I, De Bruin T, Verhoeven G, Polis I.A (2007). Comparison of tepoxalin-buprenorphine combination and buprenorphine for postoperative analgesia in dogs. J. Vet. Med.

[28] Gupta A.K, Bisla R.S, Kuldip S, Ashok K (2009). Evaluation of buprenorphine and tramadol as pre-emptive analgesics following ovariohysterectomy in female dogs. Indian J. Vet. Surg.

[29] Pibarot P, Dupuis J, Grisneaux E, Cuvelliez S, Plante J, Beauregard G, Bonneau N.H, Bouffard J, Blais D (1997). Comparison of ketoprofen, oxymorphone hydrochloride, and butorphanol in the treatment of postoperative pain in dogs. J. Am. Vet. Med. Assoc.

[30] Seliškar A, Rostaher A, Ostrouška M, Butinar J (2005). Intra-and postoperative analgesic effects of carprofen in medetomidine premedicated dogs undergoing ovariectomy. Acta Vet. Beograd.

[31] Tsai T.Y, Chang S.K, Chou P.Y, Yeh L.S (2013). Comparison of postoperative effects between lidocaine infusion, meloxicam, and their combination in dogs undergoing ovariohysterectomy. Vet. Anaesth. Analg.

[32] Vasiljević M, Ristanović D, Jovanović M, Davitkov D, Bošnjakov I, Krstić V, Stanimirović Z (2015). Comparative analysis of parameters of intraoperative and postoperative pain in bitches undergoing laparoscopic or conventional ovariectomy. Acta Vet. Beograd.

[33] Dezhang L, Chenchen W, Yupeng Y, Xinwu M (2016). Analgesic effects of lidocaine and fentanyl alone or in combination undergoing ovariohysterectomy in female dogs. Pak. Vet. J.

[34] Devitt C.M, Cox R.E, Hailey J.J (2005). Duration, complications, stress, and pain of open ovariohysterectomy versus a simple method of laparoscopic-assisted ovariohysterectomy in dogs. J. Am. Vet. Med. Assoc.

[35] Fox M.S, Mellor D.G, Lawoko C.R.O (1998). Changes in plasma cortisol concentrations in bitches in response to different combinations of halothane and butorphanol, with or without ovariohysterectomy. Res. Vet. Sci.

[36] Fantoni D.T, Ida K.K, Thais I.A, Ambrósio A.M (2015). A comparison of pre and post-operative vedaprofen with ketoprofen for pain control in dogs. BMC Vet. Res.

[37] Matteri R.L, Carroll J.A, Dyer C.J, Moberg G.P, Mench J.A (2000). Neuroendocrine responses to stress. The Biology of Animal Stress.

[38] Rudolff S.A (2011). Evaluation of Metamizole and Carprofen as Postoperative Analgesics in Canine Total Hip Replacement.

[39] Slingsby L.S, Waterman-Pearson A.E (2001). Analgesic effects in dogs of carprofen and pethidine together compared with the effects of either drug alone. Vet. Rec.

[40] Wagner A.E, Worland G.A, Glawe J.C, Hellyer P.W (2008). Multicenter, randomized controlled trial of pain-related behaviors following routine neutering in dogs. J. Am. Vet. Med. Assoc.

[41] Moberg G.P, Moberg G.P, Mench J.A (2000). Biological response to stress: Implications for animal welfare. The Biology of Animal Stress.

[42] Gutierrez-Blanco E, Victoria-Mora J.M, Ibancovichi-Camarillo J.A, Sauri-Arceo C.H, Bolio-González M.E, Acevedo-Arcique C.M, Marin-Cano G, Steagall P.V (2015). Postoperative analgesic effects of either a constant rate infusion of fentanyl, lidocaine, ketamine, dexmedetomidine, or the combination lidocaine-ketamine-dexmedetomidine after ovariohysterectomy in dogs. Vet. Anaesth. Analg.

[43] Rialland P, Authier S, Guillot M, del Castillo J.R.E, Veilleux-Lemieux D, Frank D (2012). Validation of orthopedic postoperative pain assessment methods for dogs: A prospective, blinded, randomized, placebo-controlled study. PLoS One.

[44] Mathews K.A, Pettifer G, Foster R, McDonell W (2001). Safety and efficacy of preoperative administration of meloxicam, compared with that of ketoprofen and butorphanol in dogs undergoing abdominal surgery. Am. J. Vet. Res.

[45] Roughan J.V, Flecknell P.A (2003). Evaluation of a short duration behaviour-based post-operative pain scoring system in rats. Eur. J. Pain.

